# Efficient water-related failure detection in PEM fuel cells: Combining a PEMFCs fractional order impedance model with FFT-PWM techniques and artificial neural network classification

**DOI:** 10.1016/j.heliyon.2024.e29084

**Published:** 2024-04-04

**Authors:** Fatima Zohra Arama, Slimane Laribi, Khaled Mammar, Nouar Aoun, Touhami Ghaitaoui

**Affiliations:** a Laboratoire de Développement Durable et Iinformatique (LDDI), faculté des Science et de la Technologie, Université Ahmed Draia, 01000, Adrar, Algeria; b Smart Grids & Renewable Energies Laboratory SGRE, University of Tahri Mohamed Bechar, Bp 417, Algeria; c Unité de Recherche en Energies Renouvelables en Milieu Saharien, URERMS, Centre de Développement des Energies Renouvelables, CDER, 01000, Adrar, Algeria

**Keywords:** PEMFCs, FOIM, Diagnosis, Flooding, Drying, FFT-PWM, ANN-PR, Water failure modes, FSA

## Abstract

Water management and early detection of faults in proton exchange membrane fuel cells (PEMFCs) are among the most critical constraints that limit the optimal spread of this type of energy. Consequently, it is necessary to enhance the reliability and durability of PEMFCs by developing an approach to diagnose and identify water failure modes. This paper proposes an effective and simple method to detect, diagnose, and classify various water failure modes in PEMFCs using a hybrid diagnostic approach. This approach combines the PEMFC fractional order impedance model (FOIM) with fast Fourier transform pulse width modulation (FFT-PWM) techniques and artificial neural network pattern recognition (ANN-PR) classification. The results show an accurate match between the electrochemical impedance spectroscopy (EIS) experimental data, the Nyquist impedance spectra of FOIM, and the FFT-PWM algorithm as a proposed alternative technique to EIS measurements. Learning of ANN-PR was performed using the frequency spectrum amplitude (FSA) database of the voltage and current signals produced by the PEMFCs FOIM DC/DC boost converter, which was generated using the FFT-PWM algorithm. The ANN-PR achieved low values for error accuracy, with the Low Square Error and Learning Error reaching 6.676 × 10^−19^ and 1.888 × 10^−16^, respectively. The elements inside the confusion matrix and the rest of the matrices confirm that the proposed model's accuracy, precision, recall, and high F1 score reached 100%. Furthermore, all predictions made by the ANN-PR model were consistently accurate across all areas of failure detection. Overall, the proposed method helps in analyzing, diagnosing, and classifying fuel cell failure modes such as flooding and drying, which may simplify the health assessment of PEMFC.

## Nomenclature

s: Laplace transformation
*Q*: CPE parameter or double layer capacitance at electrode/electrolyte interface (S s^α^)
*R*_*d*_: Diffusion related resistance (Ω)
*R*_*m*_: Electrolyte ohmic resistance (Ω)
*R*_*p*_: Polarization resistance (Ω)
*Z*_*T*_: Total impedance of the PEMFCs(Ω)
*Z*_*CPE*_: CPE impedance (Ω)
*Z*_*w*_: Warburg impedance (Ω)

**Greek letters**
*α*: CPE power value
*τ*_*d*_: Diffusion time constant (s)

**Abbreviations**
PWM: Pulse width modulation
FFT: Fast Fourier transform function
CPE: Constant phase elements
EIS: Electrochemical impedance spectroscopy
FSA: Frequency spectrum amplitude
RHF: Resistance at High Frequencies
EEC: Electrical equivalent circuit
FOIM: Fractional order impedance model
PEMFC: Proton exchange membrane fuel cell
FC: Fuel cell
ANN-PR: Artificial neural network pattern recognition
Ref: Reference
ROC: Receiver operating characteristic
AUC: Area under the curve

## 1 Introduction

With the world's efforts to reduce carbon emissions, attention to hydrogen energy has started to grow as a forward step in the switch to clean energy. Vehicles powered by fossil fuels are among the most significant contributors to the emission of harmful greenhouse gasses and nitrogen oxides [1,2]. Therefore, the automotive sector has invested in research and development of hydrogen proton exchange membrane fuel cells (PEMFCs) as an alternative to reduce pollution. These cells are environmentally friendly because they do not emit harmful gasses, are user-friendly, and do not produce annoying noises [3]. Despite the many advantages of hydrogen fuel cell vehicles, several challenges have prevented the spread of this type of vehicle.

These challenges include water management and early detection of failures in fuel cells (FC) (e.g., drying and flooding) [4]. To ensure optimal performance of these cells, the proton-conducting membrane must be saturated with water to ensure good ionic conductivity [5,6]. Continuous drying of the membrane leads to its deterioration; and thus, it becomes difficult to achieve good ionic conductivity. On the other hand, increased water production in the cathode may lead to blockage in gas transport at the electrodes and water saturation of the cell; therefore, it must be continuously discharged [7,8].

Lately, the detection and diagnosis of FC failure mode issues have acquired increasing attention in scientific research focusing on PEMFC water management diagnosis. Various methods for diagnosing PEMFCs can be primarily classified into approaches based on physical-mathematical models, data-driven methods, and experimental measurements [[9], [10], [11]]. The physical-mathematical approach begins by creating a mathematical performance model to study PEMFC performance under normal operating conditions. Then, the actual experimental measurements are compared with the expected values from the model to detect any errors. Mohammadi et al. [12] presented a method for modeling PEMFCs using an electrical equivalent circuit (EEC) focusing on the temperature and voltage distribution of the fuel cell. They used the Newton-Raphson method to estimate the model's physical parameters. In Ref. [13], a modified segment model was used to predict the efficiency, power distribution, and flow regime in PEMFCs under different operating conditions. In addition, the two-stage pressure drop was calculated to examine its relationship with water management diagnosis. The approach effectively diagnosed water management to determine the best cell temperature, inlet humidity, and reagent stoichiometry at different current densities.

In another study by Ren et al. [14], a semi-empirical EEC model was used to analyze the fluctuation of mass transfer during the melting process. It was found that the diffusion and dissolution resistances of the reagent increased rapidly.

The use of collected data-driven methods allows performance analysis and failure mode prediction of PEMFCs. Techniques such as voltage monitoring (I–V curve), EIS analysis, X-ray radiography, neutron radiography, and magnetic resonance measurements can be employed to detect internal system faults [[15], [16], [17], [18]]. Monitoring the voltage (or current) drop is a way to diagnose flooding and membrane drying [19].

However, this method does not allow for distinguishing between drying and flooding failures, especially at high currents, where excessive drying or flooding can lead to the same voltage drop [20,21].

Therefore, in another approach, Barbir et al. [22] used pressure drop to diagnose flooding in PEMFCs, where flooding impairs fuel access to the catalyst layer, resulting in a significant pressure drop. However, the pressure drop is not immediate for sensitive cell voltage fluctuations.

Other researchers [17,18] have used neutron radiography and magnetic resonance imaging techniques to visualize water inside PEMFCs. The use of EIS as a tool for monitoring the condition of PEMFCs is a common and useful method because of its capability to separate, identify, and isolate significantly missing cells [[23], [24], [25], [26]]. In Ref. [21], the authors focused on monitoring the water content in the fuel cell's electrolyte membrane group.

EIS measurements were used to monitor the impedance spectra evolution according to known failure conditions and to study changes in model parameters based on the stack's condition. This approach highlighted three distinct spectra based on the amount of water in the battery: one during normal operation, another during dry operation, and a third during flooding operation.

Conversely, research conducted by Tang et al. [27] suggests that measuring with EIS takes a long time, necessitating the search for rapid diagnostic techniques, especially for low-frequency flooding detection. This same vision was reaffirmed by Nahvi [28] and other researchers [[29], [30], [31], [32]], who pointed out that EIS testing exhibits high sensitivity to a variety of external conditions and internal factors occurring within FC. However, it detects significant interferences that fall in limited frequency ranges, possibly leading to difficulty in interpreting the phenomenon.

Compared with the two previous approaches, the experimental data-based approach is more flexible, efficient, and economical. These methods can analyze and use historical experimental data to create effective diagnostic models for detecting and classifying different water failure modes [33].

Accordingly, several researchers have used artificial neural network (ANN) techniques [34,35] and deep learning [36,37] to detect, diagnose, and classify of PEMFC system failures. In Ref. [38], an autonomous neural network model was introduced to diagnose faults in nonlinear PEMFC systems. Error detection includes the analysis prediction errors resulting from deviations between the model and the actual process. The simulations were conducted online by adding five different mistakes to the PEMFCs system simulator. Kahia et al. [39] used an ANN model to monitor moisture levels in an FC membrane using EIS measurements. This method facilitates the analysis and diagnosis of PEMFC failure modes, which simplifies the health status evaluation process. Kim et al. [40] employed the Hamming neural network as a pattern recognition-based diagnostic approach to identify appropriate model parameters for PEMFCs. The goal is to diagnose the state of health of these cells using the FC output voltage patterns. Benouioua et al. [41] developed a practical tool for diagnosing FC air supply systems. A simulated FC airline was used to study normal and abnormal operations by collecting data using an air pressure transducer. Pattern recognition was applied to statistical features from the pressure signal, achieving high (>95%) fault detection rates when the pressure regulation was off. Both 1 kHz and 100 Hz data logging proved effective for fault detection. Escobet et al. [42] presented a hybrid fault diagnostic method that combines fuzzy logic and pattern recognition for PEMFC power systems. Using a PEMFC nonlinear simulator with five fault scenarios, the method swiftly detects and identifies faults, outperforming traditional model-based approaches.

However, the abovementioned methods remain subject to historical experimental data, and most require relatively expensive experimental equipment. Therefore, many researchers have resorted to exploiting experimental data obtained using fast Fourier transform (FFT) analysis to address these difficulties. Consequently, this technology has been used in various areas, such as renewable energy systems [43], motor control [44], and electrochemical systems [45,46]. Indeed, this technology can solve complex waveforms, characterize individual frequency components, provide accurate signal analysis, and detect errors.

In PEMFC electrochemical systems, Chen et al. [47] studied the state and dynamics of PEMFCs using FFT methods, which effectively detect water management errors occurring at the anode or cathode. In Ref. [48], the authors proposed an online fault diagnostic method for PEMFCs using fast EIS measurements. They used a periodic triangular wave as a disturbance current, and the impedance spectrum was obtained using an FFT method. Cruz-Manzo et al. [49] proposed a computational algorithm based on FFT to evaluate the validity of EIS measurements of PEMFCs.

**Fig. 1:**
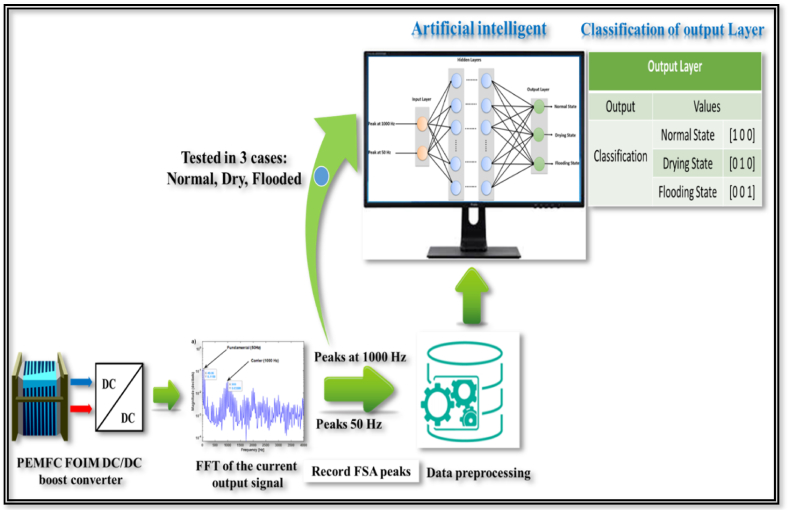
Schematic overview of the PEMFC water failure mode detection and diagnostic technique.

This study aimed to develop an effective and simple method for identifying, diagnosing, and classifying different water failure modes in PEMFCs. This approach relies on the PEMFC fractional order impedance model (FOIM) and a hybrid diagnostic method. The hybrid diagnostic method integrates fast Fourier transform pulse width modulation (FFT-PWM) techniques with an artificial neural network (ANN). The ANN is based on a neural pattern recognition model to identify faults. Fig. 1 provides a schematic overview to further illustrate the proposed approach for detecting and diagnosing water failure modes in PEMFCs.

The paper was organized as follows: initially, theoretical modeling of the PEMFCs FOIM was performed, followed by an investigation into the impact of both drying and flooding conditions on the FOIM model. The effects of water-related faults were assessed using Nyquist impedance spectra. The subsequent stage involved fault detection and diagnosis using the FFT-PWM method. This was promptly followed by data collection and the development of the ANN-PR model. Then, simulation, detection, and classification of failure were implemented using MATLAB and Simulink. Finally, the findings drawn are included in the conclusion.

## 2 Fractional-order impedance model of PEMFCs

Recently, several innovative electrical impedance models have emerged to effectively capture the intricate dynamics of PEMFCs [50,51].

**Fig. 2:**
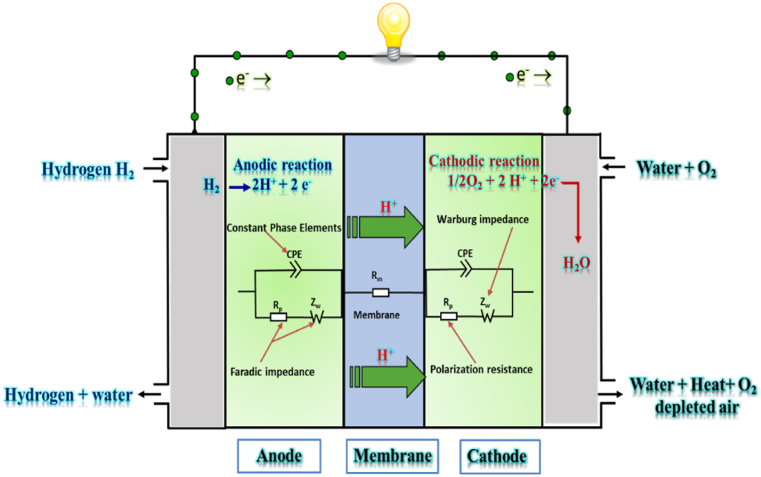
EEC of PEMFCs based on the CPE model with dissociated electrodes, including the basic PEMFC operation.

These models can remarkably anticipate the hydration states and accurately evaluate the electrical performance of PEMFCs, regardless of the operational mode (i.e. normal operation, flooding, drying). Among the many available models, the widely used and accepted approach for PEMFCs modeling is the modified Randle circuit model augmented with a constant phase element (CPE) (depicted in Fig. 2) [21,52,53].

Fig. 2 combines the PEMFC impedance model and its basic operation. This model includes two resistors, with one primarily representing the electrolytes and connector resistance (*R*_*m*_), while the other is responsible for capturing the transfer charge resistance (*R*_*p*_). In addition, the model incorporates a CPE to effectively simulate the electrical double-layer phenomenon. Lastly, the diffusion processes are characterized by an impedance *Z*_*w*_(s), which adeptly accounts for the Warburg boundary condition [54,55].

In the basic operation of PEMFCs, the cell is fed pure hydrogen and oxygen under a certain pressure from the anode and cathode. The proton products pass through the solid polymer membrane, forcing the electrons to pass through an external circuit and producing electricity [25].

In this study, the equation that encompasses the comprehensive PEMFC impedance can be expressed by equation (1) as follows:

(1) $$Z_{T}\left(s\right)=R_{m}+\left[Z_{CPE}\left(s\right)//\left(R_{P}+Z_{w}\left(s\right)\right)\right]$$

The *Z*_*CPE*_ impedance and the general expression for the Warburg impedance *Z*_*w*_(s) can be represented by the following equations (equation (2) and (3)):

(2) $$Z_{CPE}\left(s\right)=\frac{1}{Q.s^{\alpha }}$$

(3) $$Z_{w}\left(s\right)=R_{d}\frac{\text{th}\left[{\left(\tau_{d}.s\right)}^{\frac{1}{2}}\right]}{{\left(\tau_{d}.s\right)}^{\frac{1}{2}}}$$

whereas α represents a real number typically ranging between 0 and 1. The *τ*_*d*_ and *R*_*d*_ represent the time diffusion and resistance associated with the diffusion process, respectively. By incorporating equations (2), (3) into equation (1), the total impedance, as indicated by equation (1), can be reformulated by equation (4) as follows:

(4) $$Z_{T}\left(s\right)=R_{m}+\frac{1}{\frac{1}{Q.s^{\alpha }}+\left(\frac{1}{\left(R_{P}+R_{d}\frac{\text{th}\sqrt{\left(\tau_{d}.s\right)}}{\sqrt{\tau_{d}.s}}\right)}\right)}$$

The hyperbolic tangent was substituted to estimate the fractional modeling expression for the Warburg impedance [56] using equation (5), resulting in equation (6). Subsequently, after simplification, the expression for the Warburg impedance can be represented as follows.

(5) $$\left\{ \begin{array}{l} \text{th}\left(x\right)=\frac{\sinh\left(x\right)}{\cosh\left(x\right)}=\frac{\frac{e^{x}-e^{-x}}{2}}{\frac{e^{x}+e^{-x}}{2}}\underset{x\to 0}{\approx }\frac{x}{1+\frac{x^{2}}{2}}\underset{x\to 0}{\approx }\frac{x}{\sqrt{1+x^{2}}}\\ \text{th}\left(x\right)=\frac{\sinh\left(x\right)}{\cosh\left(x\right)}=\frac{\frac{e^{x}-e^{-x}}{2}}{\frac{e^{x}+e^{-x}}{2}}\underset{x\to \infty }{\approx }\frac{e^{\frac{x}{2}}}{e^{\frac{x}{2}}}\underset{x\to \infty }{\approx }\frac{x}{\sqrt{1+x^{2}}} \end{array}\right.$$

(6) $$Z_{w}\left(s\right)≅\frac{R_{d}}{\sqrt{1+\tau_{d}.s}}$$

The subsequent equation (equation (7)) is derived by employing the Taylor series expansion for the square root of the denominator in the preceding expression:

(7) $$\frac{1}{\sqrt{1+s.\tau_{d}}}=\frac{1}{{\left(s.\tau_{d}\right)}^{0.5}+0.5\times \frac{1}{{\left(s.\tau_{d}\right)}^{0.5}}+\delta \left(s.{\tau_{d}}^{2}\right)}$$

The global model for the fractional-order impedance of the PEMFCs (equation (1)) was reconfigured as equation (8):

(8) $$Z_{T}\left(s\right)=R_{m}+\frac{1}{Q.s^{\alpha }+\frac{1}{R_{p}+\frac{R_{d}}{{\left(s.\tau_{d}\right)}^{0.5}+0.5\times \frac{1}{{\left(s.\tau_{d}\right)}^{0.5}}+\delta \left(s.{\tau_{d}}^{2}\right)}}}$$

The PEMFC FOIM can be expressed as a ratio, depicted in equation (9) as follows:

(9) $$Z_{T}\left(s\right)=\frac{x_{0}+x_{1}s^{0.5}+x_{2}s^{0.8}+x_{3}s^{1}+x_{4}s^{1.3}+x_{5}s^{1.8}}{1+y_{1}s^{0.8}+y_{2}s^{1}+y_{3}s^{1.3}+y_{4}s^{1.8}}$$

The coefficients *x*_i_ and *y*_j_ in this context are dynamic functions that depend on the specific physical characteristics of the PEMFCs. These coefficients can be determined using the following equations (equation (10) and (11)).

(10) $$\left\{ \begin{array}{l} x_{1}=R_{p}Q\\ x_{2}=2\tau_{d}\\ x_{3}=2R_{d}Q{\tau_{d}}^{0.5}\\ x_{4}=2R_{p}Q\tau_{d} \end{array}\right.$$

(11) $$\left\{ \begin{array}{l} y_{0}=R_{m}+R_{p}\\ y_{1}=2R_{d}{\tau_{d}}^{0.5}\\ y_{2}=R_{m}QR_{p}\\ y_{3}=2\tau_{d}\left(R_{m}+R_{p}\right)\\ y_{4}=2R_{m}QR_{d}{\tau_{d}}^{0.5}\\ b_{5}=2R_{m}QR_{d}\tau_{d} \end{array}\right.$$

The deviation between the Warburg impedance and its approximation based on the Taylor series expansion is within a small margin of 3%. Moreover, the average value of this deviation is remarkably low at 0.09% across a broad range of frequencies [57].

**Fig. 3:**
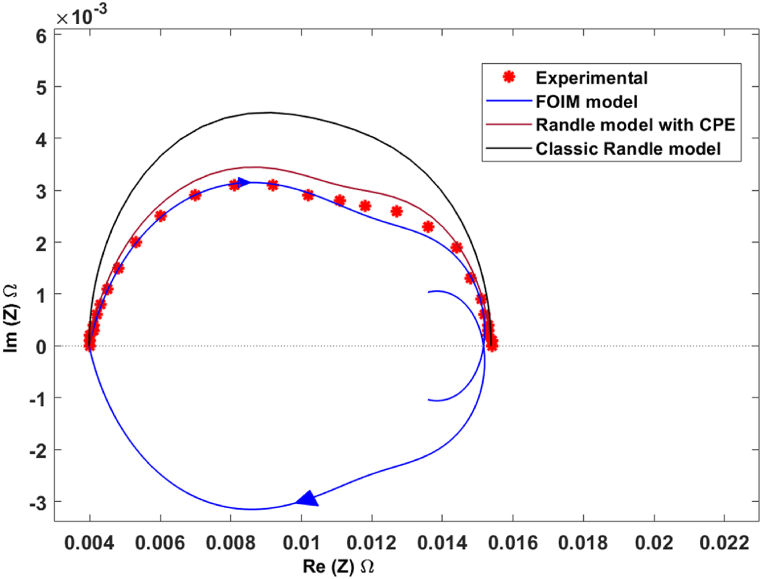
Comparison between PEMFC experimental impedance [21], FOIM, and both Randles models.

Upon comparing the experimental data from Ref. [21] with the PEMFC FOIM Nyquist diagram spectra and the Randles models, it is evident that the proposed model outperforms both Randles models, as observed in Fig. 3. In contrast, both Randles models (classic and improved with CEP) exhibit limited performance in simulating the EIS of PEMFCs, particularly in the medium frequency range.

The suggested model outperforms both conventional Randles models in that it can better represent the intricate electrochemical behavior and impedance characteristics of PEMFCs. This improved performance in the EIS simulation helps in a more in-depth understanding of the underlying mechanisms and processes within the PEMFC system. It also facilitates a better understanding and optimization of the cell's performance, which promotes advances in the diagnostic techniques of PEMFC failure modes.

**Fig. 4:**
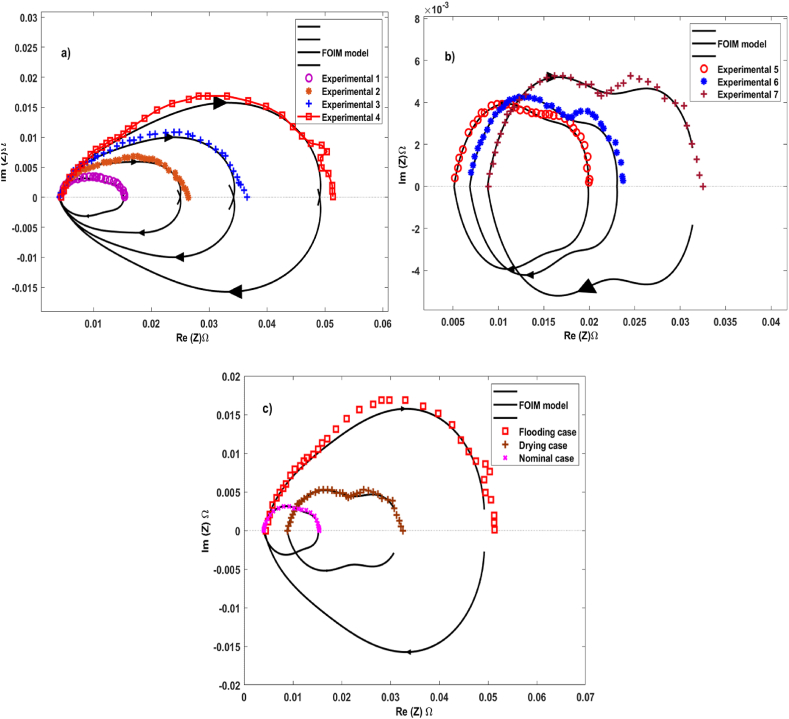
Validation of the PEMFC FOIM impedance spectrum with the experimental tests from Ref. [21] in different failure modes: a) Flooding cases; b) Drying cases; c) Comparison between three cases.

Extensive tests were conducted to confirm the efficiency of the proposed model under different fuel cell operating conditions. Fig. 4 depicts the PEMFC FOIM impedance spectrum's validation compared with empirical data from Ref. [21]. This validation includes testing for flooding, drying, and normal conditions, and a comprehensive comparison of all three scenarios. The Nyquist impedance spectra curves, represented by symbols, are plotted in the complex plane. The continuous lines correspond to the simulation curves of the PEMFC FOIM. These curves were generated after including the parameters of the corresponding EEC shown in Fig. 2. The experimental data parameters were obtained from Ref. [21] for flooding and drying cases (see Table 1, Table 2, Table 3). These results provide a better understanding of the behavior of fuel cells under different operating conditions.

**Table 1:** Physical parameters of PEMFCs [21,57].

Parameters	*R*_*m*_ (*m*Ω)	*Q*(S s^α^)	*R*_*p*_ (*m*Ω)	*R*_*d*_ (*m*Ω)	*τ*_*d*_(*m*s)
Normal	4.2	1.6	6	5	160
Drying	8.80	0.620	13	10.1	183.5
Flooding	4.16	0.936	16.3	31.2	94.7

**Table 2:** Physical parameters of PEMFCs in flooding cases [21].

Parameters	*R*_*m*_ (*m*Ω)	*Q*(S s^α^)	*R*_*p*_ (*m*Ω)	*R*_*d*_ (*m*Ω)	*τ*_*d*_(*m*s)
Experimental 1	3.98	1.109	8	3.4	87.2
Experimental 2	4.06	1.080	12.3	9.4	81.8
Experimental 3	4.00	1.102	14.7	17.2	78.4
Experimental 4	4.16	0.936	16.3	31.2	94.7

**Table 3:** Physical parameters of the PEMFC drying membrane [21].

Parameters	*R*_*m*_ (*m*Ω)	*Q*(S s^α^)	*R*_*p*_ (*m*Ω)	*R*_*d*_ (*m*Ω)	*τ*_*d*_(*m*s)
Experimental 5	5.12	0.952	9.9	5.1	115.5
Experimental 6	6.85	0.684	10.8	5.6	122.3
Experimental 7	8.80	0.620	13.0	10.1	183.5

In the flooded case (Fig. 4a), a negligible shift in the impedance spectra toward the positive real axis was observed at high frequencies. The point of intersection with the real part is commonly prescribed as the PEMFC resistance at high frequencies (RHF). At low frequencies, the impedance spectra demonstrated growth in the complex plane, with the two semicircles becoming visually indistinguishable. In contrast, in the drying case (Fig. 4b), the Nyquist impedance spectra smoothly shifted toward the right side of the real axis, exhibiting symmetrical semicircular arcs at both high and low frequencies. This behavior contrasts with the flooded and nominal conditions. In the nominal case (Fig. 4c), a distinct pattern emerged, displaying a fully depressed semicircle at high frequencies and a smaller semicircle at low frequencies.

## 3 FFT-PWM diagnostic method

The FFT-PWM diagnostic method is a powerful tool for analyzing, fault detection, and condition monitoring in various scientific and engineering domains [58,59].

**Fig. 5:**
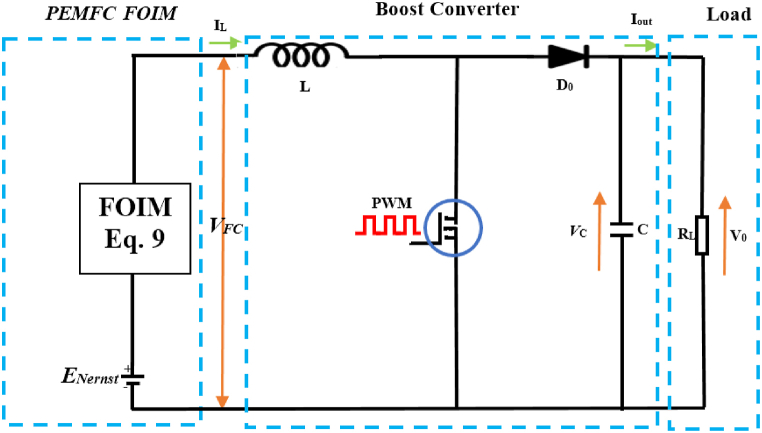
Design of the PEMFCs FOIM DC/DC boost converter.

In this method, PWM pulses are injected as perturbation waves into a DC/DC boost converter connected in parallel with the FOIM (equation (9)) of the PEMFCs, where the cell voltage (*E*_*Nerns*_) is associated (Fig. 5). Where, the PWM signal employed in the study is a square wave generated by comparing a low-frequency sinusoidal signal (*f =* 50 Hz) with a high-frequency sawtooth signal (*f* = 1 kHz). This configuration allowed the generation of precise PWM, enabling effective perturbation of the system under investigation. The application of the PWM input signal distorts the voltage and current signals, providing insights into the nonlinear processes within the system. Response frequency domain analysis was performed using the MATLAB software FFT algorithm. This analysis helps characterize higher-order harmonics [60].

The FFT-PWM method used in this research has limitations that must be considered. It assumes a linear and time-invariant system, which may not fully capture the nonlinear dynamics or time-varying characteristics of certain systems. Furthermore, the accuracy of the method is also impacted by the signal-noise ratio, the resolution of the frequency spectrum analysis, restrictions pertaining to parameter selections, and underlying assumptions. Therefore, it is better to validate the results through experimental studies to ensure the applicability of this technology in real-world environments, which is our future aspiration.

The selection of components for the DC/DC boost converter, namely, the inductor (*L*) and capacitor (*C*), was primarily influenced by factors such as the voltage ripple across the capacitor (*ΔV*_*C*_), the current ripple in the inductor (*ΔI*_*L*_), the switching frequency (*fs*), and the duty cycle (*α*_*m*_). The determination of appropriate *L* and *C* values was based on equations (12), (13), respectively [61,62]:

(12) $$L=\frac{V_{PEMFC}\alpha_{m}}{\Delta I_{L}f_{s}}$$

(13) $$C=\frac{V_{0}d_{m}}{\Delta V_{0}f_{s}R_{load}}$$

in addition, the duty cycle (*α*_*m*_) was estimated by considering the minimum voltage value of the PEMFCs denoted as *V*_*PEMFC_min*_. This minimum voltage value was represented using equation (14) [62,63]:

(14) $$\alpha_{m}=1-\frac{V_{PEMFC\_\min}}{V_{0}}$$

Table 4 presents an overview of the boost converter parameters derived from an analysis of the anticipated variations in the PEMFC operating conditions and the continuous operation mode of the DC/DC boost converter.

**Table 4:** Essential design parameters for the DC/DC boost converter [62].

N^°^	Specification parameters	Value
1	Input voltage level	5–12
2	Output voltage level	15–25
3	Max current ripple (*ΔI*_*L*_*)*	5%
4	Max voltage ripple (*ΔV*_*C*_)	5%
5	*fs*	10 kHz
6	*L*	>30 μH
7	*C*	>10 mF

On the basis of the FFT function in Matlab, the FOIM amplitudes of the PEMFCs are obtained in different operating states.

## 4 Classification of PEMFC failure modes using ANN-PR

Accurate and timely detection of failure patterns is crucial for optimizing the performance and longevity of PEMFCs. These failure modes can arise from various factors, with the hydration status of the cells having a significant influence on their efficiency and reliability. To address this challenge, the implementation of ANNs based on pattern recognition has emerged as an innovative and promising method.

**Fig. 6:**
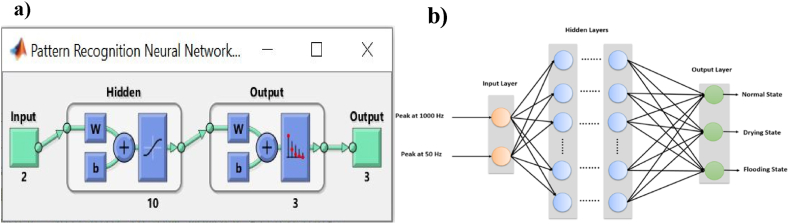
ANN-PR configuration for PEMFC failure mode detection; a) Matlab software application of ANN-PR; b) ANN-PR architecture mode.

The configuration of the neural pattern recognition model used in this research is depicted in Fig. 6. ANN-PR was applied using Matlab software (Fig. 6a). Where, the ANN-PR comprises a two-layer feed-forward network with hidden sigmoid neurons and softmax output neurons (model network). The Softmax transfer function is employed to classify neural networks. The input layer consists of two neurons: the first associated with FSA at a high frequency of P1000 Hz and the second linked to FSA at a low frequency of P50 Hz (Fig. 6b). The hidden layer contains ten neurons, and the output layer consists of three neurons, each providing values between 0 and 1. The training of the ANN-PR model used the inverse conjugated gradient (trainscg) method.

The ANN-PR database is generated, consisting of 3000 cases, by implementing the FFT function in Matlab. The FFT function is applied to the voltage and current signals produced by the PEMFCs FOIM DC/DC boost converter in section 3.

The FSA values were recorded at high and low frequencies (P1000 Hz and P50 Hz) based on changes in the PEMFC hydration state. These values serve as essential features for pattern recognition. These data include 1000 cases of PEMFC under drying conditions, with humidity levels ranging from 10% to 72%. Another 1000 cases represent the PEMFC under normal conditions, with humidity levels ranging from 73% to 84%. The remaining 1000 cases represent the PEMFC in a flooding state, with humidity levels between 85% and 100%.

Out of the dataset of 3000 cases, 70% (2100 cases) were used for training the ANN-PR, 15% (450 cases) for validation, and the remaining 15% (450 cases) for testing. This segmentation strategy ensures that the model is trained, validated, and tested on distinct subsets of the dataset.

**Fig. 7:**
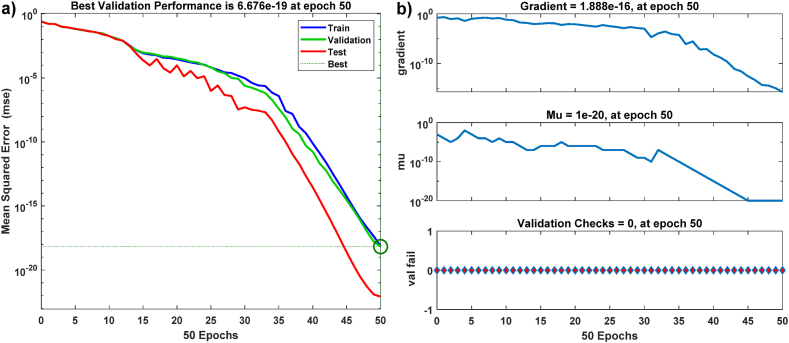
ANN-PR performance: a) ANN-PR training mean square error; b) ANN-PR output errors during learning.

Fig. 7a shows a representation of the mean squared error. After 50 iterations, ANN-PR achieves an impressively low square error value of 6.676 × 10^−19^. The network learning error is shown in Fig. 7b, and it detects a learning error close to zero (about 10^−16^), which is strong evidence of the high reliability of ANN-PR learning.

## 5 Results and discussions

### 5.1 Frequency spectral analysis and diagnosis

After comparing the experimental data from Ref. [21] with the PEMFC FOIM Nyquist diagram spectra and the Randles model, the proposed model showed remarkable superiority over the Randles model.

The proposed model (expressed by equation (9)) was implemented in a simple electrical circuit (Fig. 5) to monitor the PEMFCs hydration state (flooding, dry and normal) on FSA.

On the basis of the FFT-PWM diagnostic method, the amplitudes of the impedance frequency spectra were obtained by using the FFT function in Matlab. The FFT was applied to the voltage and current signals produced by the PEMFCs FOIM DC/DC boost converter. The physical parameter values (*R*_*m*_, *R*_*p*_, *R*_*d*_, *Q*, *τ*_*d*_) representing the flooded, dry, and normal states of the PEMFC membrane are given in Table 1, Table 2, and **3**, respectively.

**Fig. 8:**
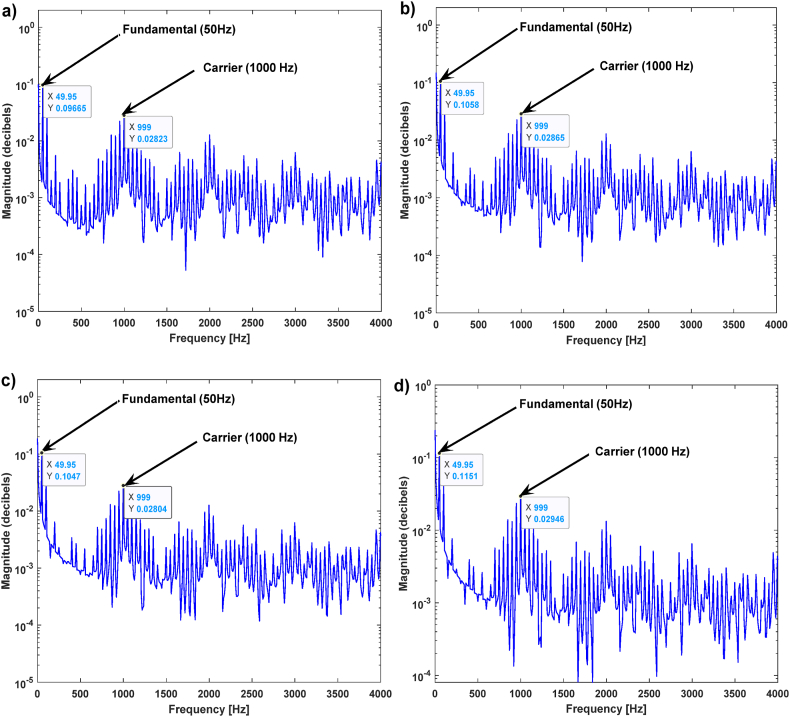
PEMFC FOIM frequency spectrum amplitude in the flooding case a) FSA of Experimental 1, b) FSA of Experimental 2, c) FSA of Experimental 3, d) FSA of Experimental 4.

Fig. 8 shows the frequency spectra of the PEMFC FOIM impedance obtained under the PEMFC flooding condition. The FSA values at high frequencies (1000 Hz) and low frequencies (50 Hz) were recorded in the PEMFC flooded state, as shown in Table 5. It can be seen from Fig. 8 and Table 5 that, at the high frequency (1000 Hz), the values of the FSA were almost constant, due to the constant RHF of the PEMFCs in the flooding state. At the lower frequency (50 Hz), there was a variation in the FSA values because the measured polarization resistance of the PEMFCs varied as well. These observations are confirmed by the experimental results and simulations shown in Fig. 4a. It was noted that the RHF is constant at high frequencies, and the measured polarization resistance is variable at low frequencies.

**Table 5:** Extracted results from the PEMFC flood failure mode simulations.

Shown in	FSA values	
	Peak at 50Hz	Peak at 1000 Hz
Fig. 8a (PWM FFT of Experimental 1)	0.09665	0.02823
Fig. 8b (PWM FFT of Experimental 2)	0.1058	0.02865
Fig. 8c (PWM FFT of Experimental 3)	0.1047	0.02804
Fig. 8d (PWM FFT of Experimental 4)	0.1151	0.02946

**Fig. 9:**
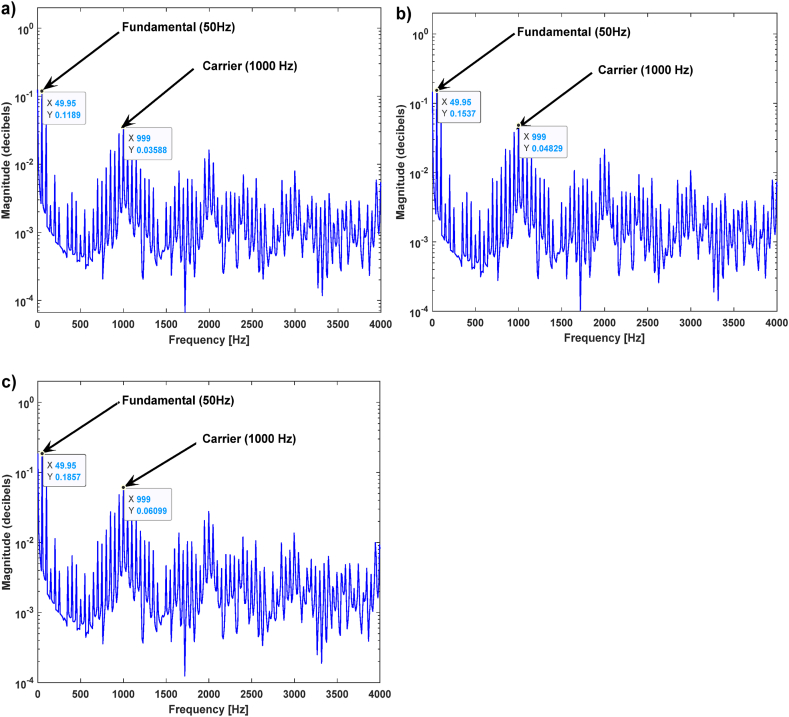
PEMFC FOIM frequency spectrum amplitude in the drying case. a) FSA of Experimental 1, b) FSA of Experimental 2, c) FSA of Experimental 3.

Fig. 9 shows the frequency spectra of the PEMFC FOIM impedance in the fuel cell drying state. The FSA values at high (1000 Hz) and low (50 Hz) frequencies were recorded in the dry state (Table 5). From Fig. 9 and Table 6, at the high frequency (1000 Hz), the values of the FSA varied due to the change in the PEMFCs RHF in the drying state. Simultaneously, at the lower frequency (50 Hz), there was a variation in the FSA because the measured polarization resistance of the PEMFCs also varied. These observations are confirmed by the experimental results and simulations shown in Fig. 4b. It was noted that the measured RHF and polarization resistance were variable at high and low frequencies, respectively.

**Table 6:** Extracted results from the PEMFC dry failure mode simulations and nominal case.

Shown in	FSA values	
	Peak at 50Hz	Peak at 1000 Hz
Fig. 9a (PWM FFT of Experimental 5)	0.1189	0.0588
Fig. 9b (PWM FFT of Experimental 6)	0.1537	0.0829
Fig. 9c (PWM FFT of Experimental 7)	0.1857	0.06099
Fig. 10 (PWM FFT of Nominal Experimental case)	0.0826	0.02855

**Fig. 10:**
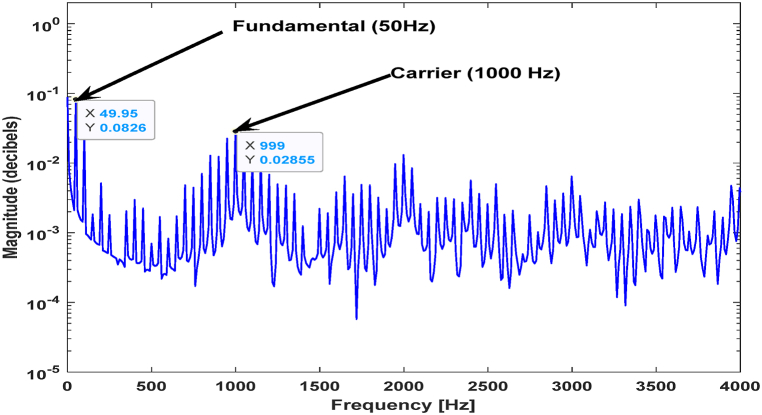
PEMFC FOIM frequency spectrum amplitude under normal conditions.

Fig. 10 shows the impedance frequency spectrum of a PEMFC FOIM for a normal fuel cell.

From Fig. 10 and Table 6, at the high frequency (1000 Hz), the values of the FSA were almost constant in the normal and flooding conditions, whereas they were variable in the drying case. This is because the RHF increases in the drying case and is nearly constant in the flooding case. At the same time, there was a variation in the FSA at the lower frequency (50 Hz) because the measured polarization resistance of the PEMFCs varied as well. These observations are confirmed by the experimental results and simulations shown in Fig. 4c. It was noted that the RHF is almost constant under the normal and flooding conditions. However, it increases in the drying state, but at low frequencies, the measured polarization resistance varies.

### 5.2 ANN-PR application for identifying PEMFC failure patterns

Having verified the high reliability of the ANN-PR model's learning, as evidenced by the low value of squared error and a learning error close to zero, this model is used to detect the failure modes of PEMFCs.

**Fig. 11:**
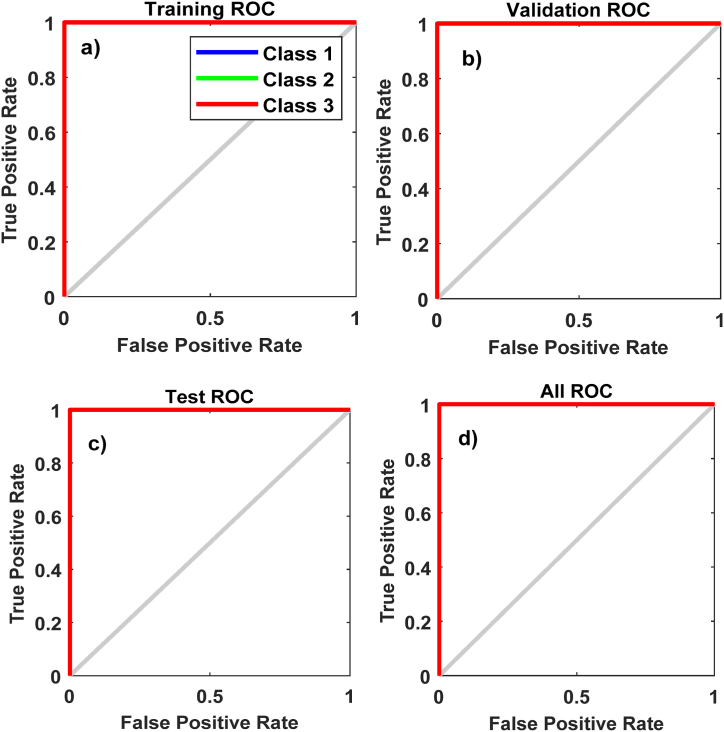
ANN-PR training ROC curves; (a) Training ROC; (b) Validating ROC; (c) Test ROC; and (d) All ROC.

Fig. 11 shows the receiver operating characteristic (ROC) curves used to determine the overall performance of the module. It is noted that the proposed ANN-PR model is excellent because the area under the curve (AUC) is near 1 (Fig. 11a), which means that it has a good measure of reparability.

An identical trend was observed in the remaining ROC curves for ANN-PR training: the validation ROC (Fig. 11b), testing ROC (Fig. 11c), and overall ROC (Fig. 11d). The outcomes confirm that the proposed model is appropriate for categorizing PEMFC failure scenarios.

**Fig. 12:**
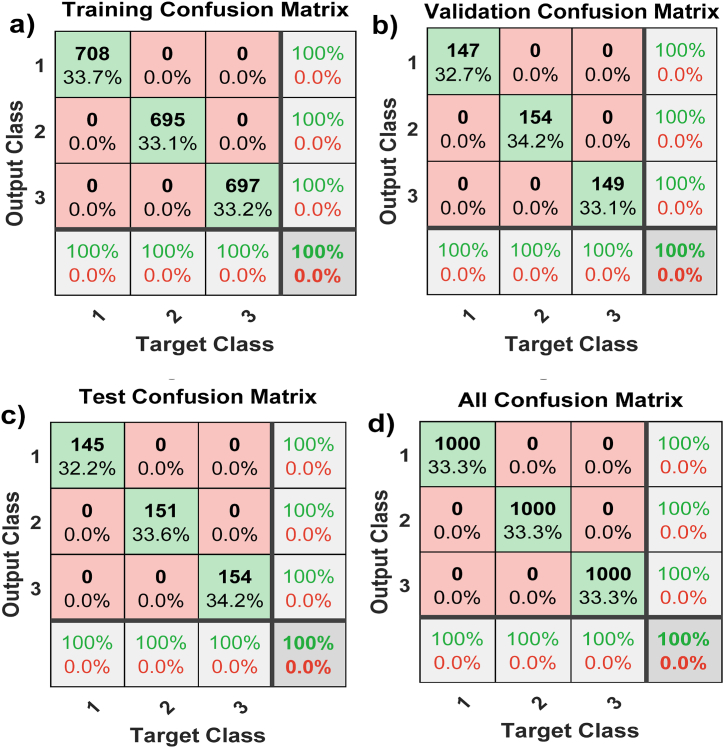
ANN-PR training confusion matrices. (a) Training confusion matrix; (b) Validating confusion matrix; (c) Test confusion matrix; and (d) All confusion matrix.

Fig. 12 presents the ANN-PR confusion matrix plot. This tabular representation summarizes the results of the diagnostic algorithm's predictions regarding the nominal condition and PEMFC failure patterns, providing a comprehensive overview of the model's performance.

In Fig. 12a, the cells along the main diagonal represent the number and proportion of correct classifications made by the trained network. For example, 708 cases of PEMFCs were accurately classified as being in normal condition. This represents 33.7 % of the total 2100 cases allocated to ANN-PR training. Similarly, 695 PEMFC cases were correctly classified as being in dry condition, constituting 33.1% of all cases. Likewise, 697 PEMFC cases were precisely classified as being in the flood state, which is 33.2% of all cases. Moreover, there were no droughts or floods in the PEMFCs that were erroneously classified as normal. This represents 0% of all cases in the dataset.

Similarly, no occurrences of the normal case were inaccurately classified as either dry or flooded, corresponding to 0% of the entire data set. In addition, among the 708 predictions of the normal state, 100% were accurate, and there were no false predictions, resulting in an error rate of 0%. Similarly, for the 695 dry-state predictions, all are correct, with 0% being false. Among the 697 predicted flood states, all were correctly identified as flood conditions, whereas none were misclassified as normal or dry.

Overall, all predictions were correct, producing an accuracy rate of 100%, and no forecasts were incorrect, resulting in a 0% error rate. In addition, the elements within the confusion matrix affirm that the accuracy, precision, recall, and high F1-score of the proposed model all stand at 100%. This reinforces that all predictions made by the ANN model are consistently accurate across the board. The model successfully balanced precision and recall, with a minimal number of positive and negative false values.

The same pattern applies to the remaining confusion matrix figures related to neural network training: the validating confusion matrix (Fig. 12b), test confusion matrix (Fig. 12c), and all confusion matrix (Fig. 12d). The results confirm the suitability of the proposed model for classifying PEMFC failure modes.

Following validation of the high efficiency of the ANN-PR model for detecting and classifying multiple faults in PEMFC systems, this model is used in the MATLAB Simulink software in the black box form with two inputs. The first input captures the peak FSA values at high frequencies (1000 Hz), and the second captures the peak FSA values at low frequencies (50 Hz). The outputs of the ANN-PR model include three outputs, each with a value of either zero or one. The first output predicts the normal state of the PEMFCs, the second the dry state, and the third the flooding state **(see** Table 7**).**

**Table 7:** Testing an ANN-PR model for classification in different water failure modes.

ANN-PR Inputs		ANN-PR Output	PEMFC State
Peak at 50 Hz	Peak at 1000 Hz		
**0.0826**	0.02855	[1 0 0]	Normal
**0.1189**	0.0588	[0 1 0]	Drying
**0.1537**	0.0829		
**0.1857**	0.06099		
**0.09665**	0.02823	[0 0 1]	Flooding
**0.1058**	0.02865		
**0.1047**	0.02804		
**0.1151**	0.02946		

The ANN-PR model was tested using applied tests drawn from the experiments of Fouquet et al. [21]. These tests were not previously part of the training data, which provided greater credibility to the model's performance.

The FSA input values of the ANN-PR were obtained at high frequencies (1000 Hz) and low frequencies (50 Hz) using the FFT-PWM diagnostic method (Table 5, Table 6).

Table 7 exhibits a clear pattern where the ANN-PR model accurately predicts a flood state when using the FSA values as input parameters (Table 5) at high frequencies (1000 Hz) and low frequencies (50 Hz).This alignment with the experimental findings of Fouquet et al. [21] is further supported by their traditional EIS method results shown in Fig. 4a.

Similarly, Table 7 further shows the effective prediction of the ANN-PR model in the PEMFC drying state. This prediction aligns with the outcomes of Fouquet et al. [21] based on the conventional EIS method, as shown in Fig. 4b.

The ANN-PR model's capabilities also extend to predicting the normal case (Table 7). When inputted with FSA values at high (1000 Hz) and low (50 Hz) frequencies, the model successfully identifies a normal condition for the PEMFCs. This agrees with the findings of Fouquet et al. [21] by utilizing the conventional EIS method, as displayed in Fig. 4c.

### 5.3 Summary of the comparative approaches

Table 8 includes a comparison of the computation cost of the proposed method with the EIS method. This table compares the computational requirements of the two methods. It includes relevant metrics such as processing time, memory usage, and other relevant factors contributing to computation cost.

**Table 8:** A comparative summary of the computation cost of the proposed method and the EIS method.

Computation Cost Comparison	Proposed Method	EIS Method
Processing Time	Low	High
Memory Usage	Low	High
Computational Complexity	Low	High
Algorithm Efficiency	High	Medium
Data Preprocessing	Minimal	Extensive
Accuracy	High	Medium
Scalability	Good	Limited
Robustness	High	Medium
User-Friendliness	Easy	Moderate
Energy Efficiency	Energy-efficient	High power consumption
Sensitivity Analysis	Low sensitivity	Moderate sensitivity

The use of ANN-PR based on the FFT-PWM method to diagnose and predict various water failure modes in PEMFCs applied to the voltage and current signals of the PEMFCs FOIM DC/DC boost converter presents a promising alternative. Generally, this method allows for rapid determination of the PEMFC conditions, unlike the traditional EIS method, which requires considerable time and cost.

## 6 Conclusion

In this paper, an effective and reliable technique for detecting, classifying, and diagnosing hydration-related failures in PEMFCs was developed. A hybrid diagnostic approach was employed, combining PEMFC FOIM with FFT-PWM techniques and ANN pattern recognition classification. The impact of water management conditions (normal, dry, and flooded) on the PEMFC FOIM model was thoroughly examined.

A comparison of the results between the PEMFC FOIM Nyquist impedance spectra, experimental data from Electrochemical Impedance Spectroscopy (EIS), and frequency spectrum amplitudes obtained via the FFT-PWM method demonstrated a high level of accuracy and correspondence. The ANN pattern recognition algorithm, a subset of artificial intelligence techniques, was used for fault classification. The model underwent training with FSA data extracted using the FFT-PWM algorithm from voltage and current signals produced by the PEMFCs FOIM DC/DC boost converter. The ANN-PR model's learning process exhibited high reliability, as evidenced by a low squared error value and a learning error close to zero.

The proposed model was developed by using the Matlab tools and Simulink. Evaluation tools, such as ROC curves, confusion matrices, verification curves for best performance, and error histograms have produced results with a high accuracy of 100% in simulations.

Furthermore, the proposed model was tested on three PEMFC cases (flooded, dry, and normal), and its performance was compared with experimental data from the literature [21]. These tests confirmed that the proposed ANN-PR model can detect errors rapidly with high performance and reliability.

Overall, the obtained results confirm the importance of the proposed approach in addressing the obstacles to detecting, predicting, and classifying failure modes of PEMFCs, thus contributing to saving cost, time, and maintenance efforts.

## Funding statement

This research did not receive any specific grant from funding agencies in the public, commercial, or not-for-profit sectors.

## Data availability

The data underlying this article will be shared upon a reasonable request made to the corresponding author.

## CRediT authorship contribution statement

**Fatima Zohra Arama:** Writing – review & editing, Validation, Software, Project administration, Investigation, Formal analysis, Conceptualization. **Slimane Laribi:** Writing – review & editing, Writing – original draft, Visualization, Validation, Supervision, Software, Resources, Project administration, Methodology, Investigation, Funding acquisition, Formal analysis, Data curation, Conceptualization. **Khaled Mammar:** Supervision, Data curation, Methodology. **Nouar Aoun:** Writing – review & editing, Visualization, Supervision. **Touhami Ghaitaoui:** Software, Funding acquisition, Data curation.

## Declaration of competing interest

The authors declare that they have no known competing financial interests or personal relationships that could have appeared to influence the work reported in this paper.
